# Focused ultrasound for the treatment of bone metastases: effectiveness and feasibility

**DOI:** 10.1186/s40349-018-0117-3

**Published:** 2018-11-30

**Authors:** Anne-Sophie Bertrand, Antoine Iannessi, Romain Natale, Hubert Beaumont, Sebastien Patriti, Jiang Xiong-Ying, Guillaume Baudin, Antoine Thyss

**Affiliations:** 1 Department of Interventional Radiology, Centre de Lutte contre le Cancer Antoine Lacassagne, 33 Avenue de Valombrose, 06100 NICE, France; 2 Department of Radiotherapy, Centre de Lutte contre le Cancer Antoine Lacassagne, 33 Avenue de Valombrose, 06100 NICE, France; 3 Department of Statistics, University of Nice Sophia Antipolis, 28 Avenue Valrose, 06000 NICE, France; 4 Department of Oncology, Centre de Lutte anti-Cancer Antoine Lacassagne, 33 Avenue de Valombrose, 06100 NICE, France

**Keywords:** High-intensity focused ultrasound, Bone neoplasms, Radiotherapy, Pain, Cancer

## Abstract

**Background:**

To evaluate the effectiveness and feasibility of high-intensity focused ultrasound (HIFU) for the treatment of bone metastases.

**Methods:**

A single-center prospective study was made involving 17 consecutive patients with symptomatic bone metastases. Patients were treated by Focused Ultrasound (FUs) performed with magnetic resonance (MR) guidance. Surgical treatment or radiotherapy treatment was not indicated for patients who underwent FUs. Lesions were located in the appendicular and axial skeleton and consisted of secondary symptomatic lesions. The clinical course of pain was evaluated using the Visual Analog Scale (VAS) before treatment, at 1 week, and at 1 month after treatment and the Oral Morphine Equivalent Daily Dose (OMEDD) was also recorded. We used Wilcoxon signed rank test to assess change in patient pain (R CRAN software V 3.1.1).

**Results:**

We observed a significant decrease in the pain felt by patients between pre- procedure and 1 week post-procedure (*p* = 2.9.10–4), and pre-procedure and 1 month post-procedure (*p* = 3.10–4). The proportion of responders according to the International Bone Metastases Consensus Working Party was: Partial Response 50% (8/16) and Complete Response 37.5% (6/16).

**Conclusions:**

HIFU under MR-guidance seems to be an effective and safe procedure in the treatment of symptomatic bone lesions for patients suffering from metastatic disease. A significant decrease of patient pain was observed.

**Trial registration:**

NCT01091883. Registered 24 March 2010. Level of evidence: Level 3.

## Background

For 50 years, high-intensity focused ultrasound (HIFU) has been a subject of interest for medical research [1]. HIFU triggers selective tissue necrosis in a very well-defined volume, at a variable distance from the transducer, through heating or cavitation [2]. Its potential as a non-invasive thermal ablation treatment, using real-time imaging (magnetic resonance or ultrasound) for target definition, treatment planning and closed-loop of energy deposition, has been utilized in many settings including the treatment of tumors of the liver, kidney, breast, uterus, pancreas, bones and for the relief of chronic pain of malignant origin [3–5].

Thanks to Magnetic Resonance Imaging (MRI) guidance, real-time thermal feedback of heated zones makes it possible to ablate targeted tissue in real time without damaging normal structures. The precision of the technique and the immediate feedback obtained make it an attractive and safe alternative to surgical or radiation therapy for both benign and malignant tumors [6]. Clinically, the sites accessible for HIFU treatment are limited by the need for a suitable wide and naturally available acoustic window [7]. Traditionally, HIFU treatment consists of multiple single focal point sonifications [8, 9]. In volumetric ablation, the focal spot is electronically steered along multiple concentric circles of increasing diameter and is thus more energy-efficient than point by point ablation [10]. In 2011, the Magnetic Resonance-guided Focus Ultrasound (MRgFUS) system received the European Compliance (CE) marking for the treatment of painful bone metastases [4].

Pain due to bone metastases is a common clinical problem in cancer patients [11]. The primary palliative treatment for patients with painful bone metastases is external beam radiation therapy, which achieves effective pain control in around 60–74% of patients [12–14]. More than 40% of patients are still not controlled after a second course of irradiation [15].

Magnetic resonance-guided high intensity focused ultrasound (MR-HIFU) has recently emerged as an effective treatment option for painful bone metastases by means of periosteal nerve-ending ablation. However, there exist few articles in the literature related to HIFU for this indication [4, 16–19].

The objective of our study was to describe our experience in the treatment of painful bone metastases using volumetric MR-HIFU ablation and to assess the technical feasibility and safety of the procedure [20].

## Materials and methods

### Patient population and selection

We present a prospective observational study on 17 consecutive patients (seven males, ten females, mean age: 61 years) suffering from symptomatic bone metastases of the appendicular skeleton. From October 2012 to March 2018, 17 patients with metastatic disease were enrolled. Most patients were suffering from intense inflammatory pain, often associated with mechanical pain and disability for walking or standing, depending on the localization of lesions. The lesions were located in the appendicular skeleton, involving the tibial diaphysis (two cases), femoral diaphysis (two cases), iliac bone (four cases), clavicle (one case), scapula (one case), humerus (one case), and in the axial skeleton, involving the ribs (six cases). All patients had exhausted maximum radiotherapy and analgesic treatment options for their painful bone metastasis.

For inclusion, the pain arising from the lesion had to be self-rated by the patient as ≥5 on an 11-point numeric visual analog scale (VAS) from 0 (no pain) to 10 (worst imaginable pain) [21]. Exclusion criteria were the presence of > 3 painful bone metastases, metastases located in the spine, sternum, or skull, contraindications to MR imaging or procedural sedation and analgesia (PSA), presence of a potentially unstable fracture at the site of the lesion, and lesion inaccessibility (≤1 cm distance between the lesion and major nerves, joints, blood vessels or organs) [16].

Pretreatment magnetic resonance imaging (GE Healthcare MRI 1,5 Tesla, Milwaukee, WI) was available for all patients in order to confirm the location of the bone metastases. MR images were evaluated by a radiologist with 10 years’ experience to determine treatment accessibility of the target lesion. Final treatment eligibility was determined in a multidisciplinary setting. The MRI protocol included T1-weighted (T1W) turbo spin echo (TSE) and T2-weighted (T2W) sequences in two orientations and fat-suppressed T1W (SPIR) sequences in two orientations after intravenous administration of gadobutrol, a gadolinium-based contrast agent (Gadovist, Bayer Pharma AG, Berlin, Germany, 0.1 mmol/kg). Approval was obtained from the institutional review board of the Centre Antoine Lacassagne (Nice, France) and written informed consent for the treatment and for the use of their anonymized data for this study was obtained from each patient.

### MR-HIFU system ablation

Treatments were performed by an interventional radiologist (with 5 years’ experience) using the MR-HIFU system (ExAblate 2000® MRgFUS system, Insightec, Israel). All procedures were performed under general anesthesia.

**Fig. 1:**
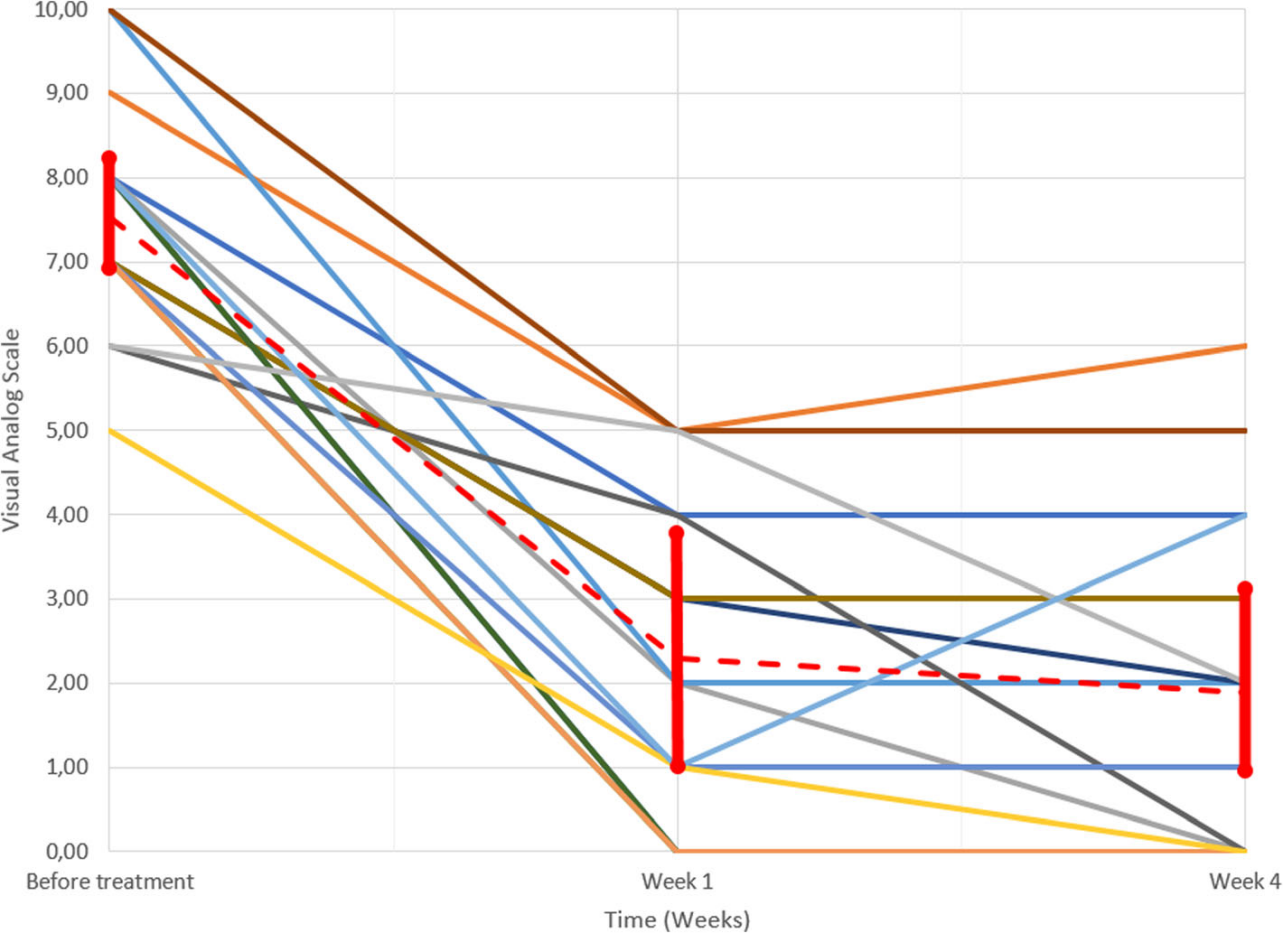
Longitudinal assessment of patient pain. Assessments were performed before treatment, after 1 week and after 4 weeks according to the Visual Analog Scale (VAS). Colored solid lines correspond to the 17 patients who were followed up. Dashed red line corresponds to the average VAS score where 95% confidence intervals are attached at each time point

Patients were placed in a prone or supine position depending on the lesion location in order to be as close as possible to the transducer for optimal treatment. After identification of the target lesion, sonications were delivered to the patient, with a number of sonications depending on the lesion size, and were adjusted in real time using temperature control at the lesion site (Fig 1).

The number of sonications delivered in our study ranged between 8 (smallest lesion) and 27 (biggest lesion). The duration of each sonication was 15 s. The average duration of the entire procedure was 2 h.

An immediate post-operative MRI was made at the end of each procedure to evaluate bone metastasis destruction, including T1-weighted fat-suppressed sequence after gadolinium injection. The non-enhanced area after treatment corresponded to the necrotic zone.

### Follow-up and response assessment

A prospective follow-up was done, consisting of post-operative evaluations at 1 week and at 1 month to assess the pain felt by the patients. Quantification of pain was made by each subject on an 11-point numeric visual analog scale (VAS) with values from 0 to 10 (where 10 indicates the strongest pain ever experienced and 0 indicates absence of pain) and was supervised by an independent evaluator. Pain evaluation was made specifically on the anatomical site treated by focused ultrasound (if there were other pain sites, they were not taken into account). A difference in VAS > 2 points was considered a clinically significant result [21]. We also recorded the oral morphine equivalent dose (OMEDD) before treatment and 1 month after treatment.

Partial response was defined as either a ≥ 2-point decrease in VAS at the treated site with no increase in OMEDD or as an OMEDD reduction of 25% or more from baseline without an increase in pain. Complete response was defined as a pain score of 0 at the treated site with no simultaneous increase in OMEDD. Pain progression was defined as a ≥ 2-point increase at the treated site with stable OMEDD or an increase of 25% or more in OMEDD compared with baseline with the pain score stable or 1 point above baseline [16]. A clinical examination was made before treatment and at 1 week and at 1 month after treatment to evaluate pain evolution.

### Statistical analysis

The VAS score was measured at these three follow-up examinations. OMEDD was recorded at baseline and after 1 month. Pre-and post-operative VAS and OMEDD were compared using the non-parametric Wilcoxon signed-rank test for paired data. *P* < 0.05 was considered statistically significant. Confidence intervals were computed by bootstrapping data. Statistical analyses were performed using R CRAN Software (Version 3.1.1).

## Results

Data are summarized in Table 1.

**Table 1:** High-intensity focused ultrasound for treatment of bone metastases: population results and technical parameters

Population								Results					Technical features^a^				
Patients	Age (years)	Sex	Primary tumor	Location	Lesion dimensions^a^ (mm)	Soft tissue invasion	Previous Radio-therapy	VAS Before	VAS D7	VAS M1	OMEDD Before	OMEDD M1	Number of Sonications	Mean Acoustic Power	Mean Temperature Monitored	Total Delivered Energy	Treated Volume (Threshold 70°)
N° 1	55	M	Larynx	Tibial diaphysis	39*38*50	Yes	No	8	4	4	30	30	17	90	60	21,400	21.2
N° 2	54	F	Breast	Femoral neck	18*14*28	No	No	9	5	6	30	0	12	50	80	18,353	4.2
N° 3	57	F	Lung	7th Rib	11*7*6	No	No	8	2	0	40	0	15	75	70	12,313	6
N° 4	78	F	Breast	Clavicle	44*29*23	No	Yes	7	3	3	60	10	26	58	75	31,763	27.1
N° 5	74	F	Endometria	Ischio-branch	50*34*33	Yes	No	10	2	2	120	10	15	152	75	73,473	27.1
N° 6	49	M	Kidney	Scapula	15*12*15	No	No	7	0	0	30	30	12	120	80	31,417	22.9
N° 7	89	M	Lung	6th Rib	9*7*5	No	Yes	7	3	2	Non-opiod	Non-opiod	40	40	60	44,244	14.9
N° 8	65	F	Lung	Humerus	32*20*27	No	Yes	10	5	5	160	160	10	100	80	20,057	17.8
N° 9	70	F	Breast	10th rib	19*7*7	No	Yes	6	4	0	300	480	7	70	80	8567	11.4
N° 10	58	F	Lung	Ilio-branch	46*34*24	Yes	Yes	7	3	3	120	120	26	120	80	75,995	44.9
N° 11	64	F	Lung	8th rib	24*13*11	Yes	Yes	8	0	0	1500	400	14	100	80	32,675	30.3
N° 12	50	F	Breast	Iliac bone	51*24*50	Yes	Yes	8	0	0	1600	240	11	220	85	47,423	43.7
N° 13	46	M	Kidney	Tibial diaphysis	29*22*47	Yes	Yes	7	1	1	60	60	9	90	75	16,875	12.4
N° 14	47	M	Lung	4th rib	35*18*32	Yes	No	7	0	0	120	120	6	60	100	8145	13.6
N° 15	60	F	Breast	T5 Transverse apophysis	13*15*20	No	Yes	6	5	2	60	120	12	80	75	20,525	11.6
N° 16	79	M	Prostate	Iliac bone	60*40*36	Yes	No	5	1	0	80	40	9	200	85	32,919	21.1
N° 17	48	M	Lung	Femoral diaphysis	40*26*60	No	No	8	1	4	20	0	16	80	70	24,764	24.2

Soft tissue invasion and lesion dimensions given as long axis (transverse) × short axis (transverse) × craniocaudal dimension (coronal) were measured at pretreatment MRI. Visual Analogic Scale (VAS) before, after 7 days and 1 month were evaluated on a 0–10 scale. Oral Morphine Equivalent Daily Dose (OMEDD) in mg before and after 1 month. Mean acoustic power in Watts. Mean Temperature Monitored in Celsius. Total Delivered Energy in Joules. Treated volume in cubic centimeters using a threshold of 70° in milliliters. ^a^Technical features were collected after treatment inside the HIFU system

### Procedure

**Fig. 2:**
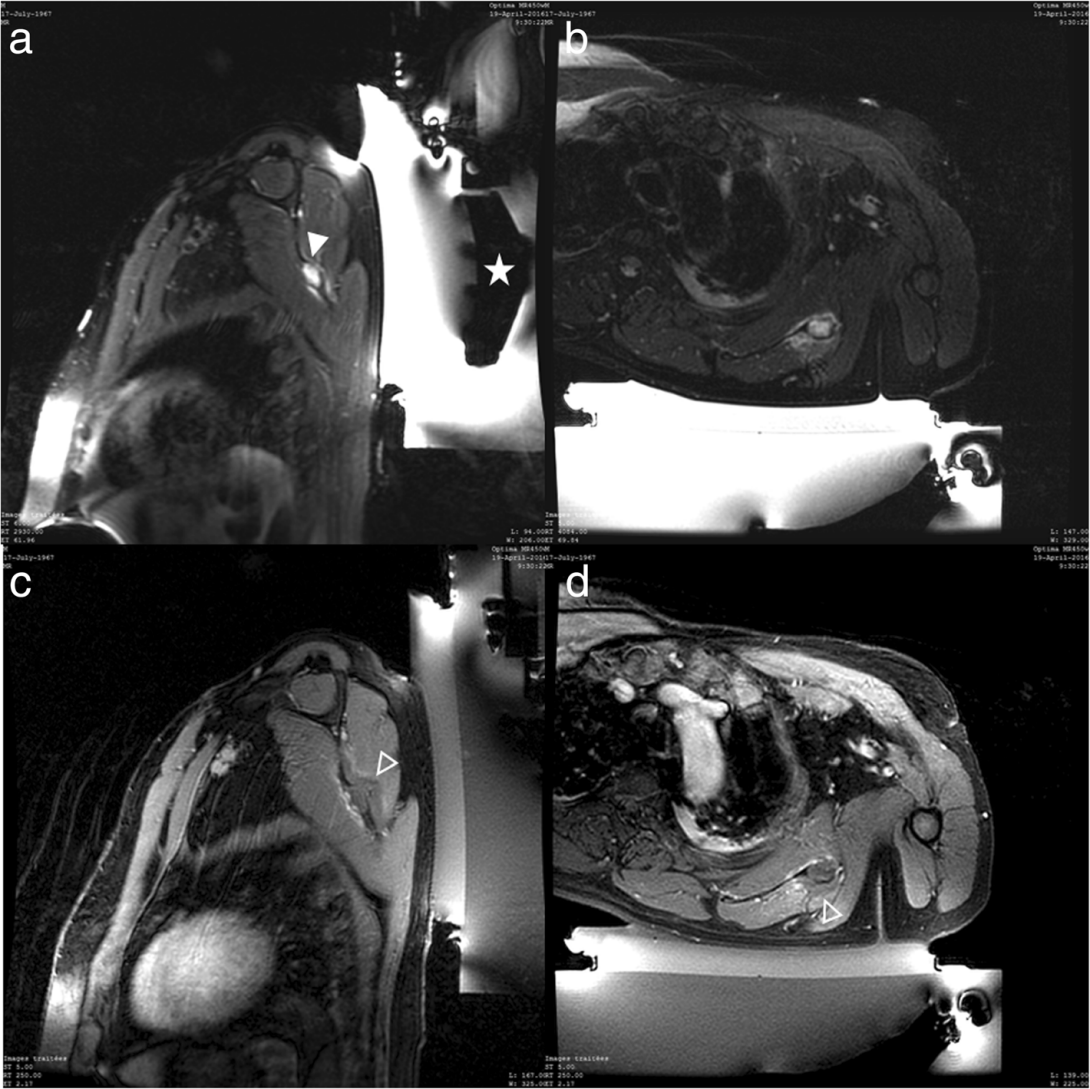
Patient 6. **a** Sagittal T2-weighted fat-suppressed MR images showing a bone metastasis of the scapula (arrowhead) in hypersignal in front of the transducer (star) before the procedure; **b**, **c**, **d** Axial T2, Sagittal and Axial T1-weighted fat-suppressed with contrast MR images after the procedure. The non-enhanced area (hollow arrowhead) is larger than the lesion and is clearly visible after the injection and corresponds to the zone of thermal destruction

**Fig. 3:**
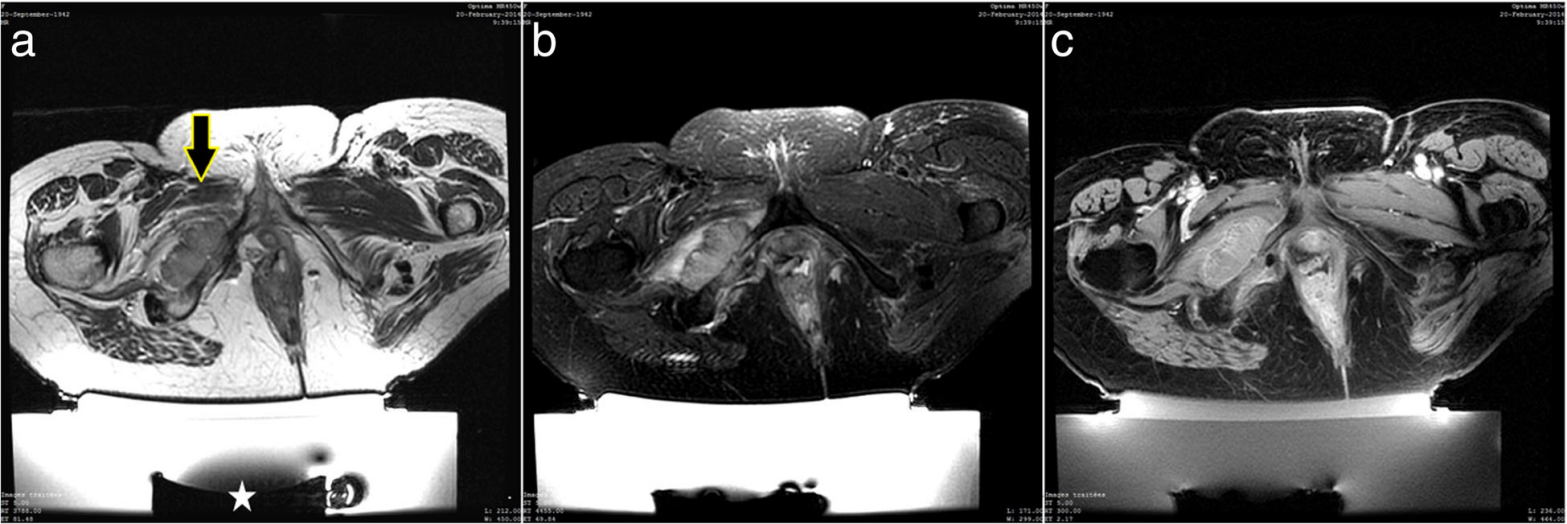
Patient 5. **a** Axial T2-weighted MR image showing a bone metastasis of the pubic ilio-branch invading adjacent soft tissues (arrow) in front of the transducer (star) before the procedure; **b**, **c**. Axial T2-weighted fat-suppressed and T1-weighted fat-suppressed with contrast MR images showing partial destruction of the lesion after the procedure

**Fig. 4:**
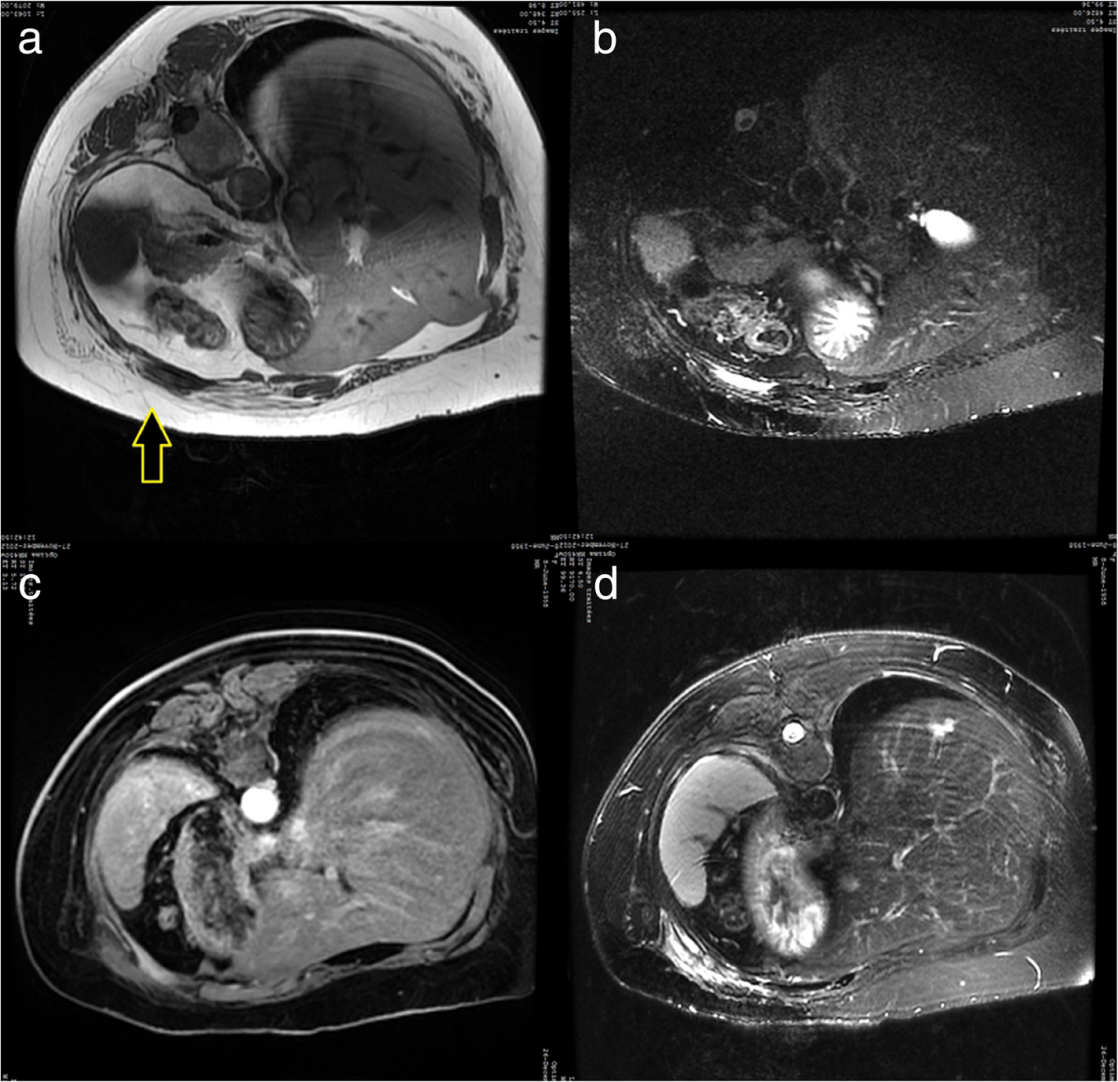
Patient 3. **a**, **b** Axial T1-weighted and T2-weighted fat-suppressed MR images showing a bone metastasis of the 7th rib before the procedure (prone position); **c**, **d**. Axial T1 with contrast MR images and T2-weighted fat-suppressed showing the lesion after the procedure

Treatment was technically successful in 17 cases and clinically successful in 16 cases according to VAS (Fig 1). After the MR-HIFU procedure, all lesions were totally or partially destroyed (Figs 2, 3, 4). The feasibility of the procedure was 100% in our study. One case was recorded in which the patient experienced no relief of his pain following the procedure. We observed no immediate or delayed complications, in particular no skin burns.

One patient received no morphine treatment and was not reported by OMEDD.

### Follow-up

Compared to the onset of treatment, all patients experienced a decrease in pain after 1 week and after 1 month. More than 40% of the cohort reported no pain at all after 1 month.

The average evaluation of pain was 7.53/17 (SD: 1.33) before treatment, 2.29/17 (SD: 1.86) 1 week after treatment, and 1.88/17 (SD: 1.99) 1 month after treatment. Our results show a significant decrease of the pain felt by patients between before the procedure and 1 week following the procedure- (*p* = 2.94.10^− 4^), and before the procedure and 1 month following the procedure- (*p* = 2.99.10^− 4^).

All patients, except patient 2, had decreasing or stable pain between 1 week and 1 month.

Sixteen of the 17 patients were satisfied with their long-lasting result following the procedure and would recommend the intervention to relatives.

Mean OMEDD was respectively 270.6 [78.31; 2293.9] at baseline and 113.75 [44.9; 270.0] at 1 month; no significant difference was observed (*p* = 0.18).

Four patients died several weeks after treatment due to their primary diseases.

### Response analysis

The proportion of responders according to the International Bone Metastases Consensus Working Party [12] was: Partial Response 50% (8/16) and Complete Response 37.5% (6/16). Overall response was 87.5% [62, 98%].

Progression of pain was observed in 12.5% (2/16) of patients. One patient who experienced pain progression was documented to have not been properly treated for pain. The other patient experiencing pain progression had a VAS score of zero but had a significantly higher OMEDD after 1 month.

## Discussion

To our knowledge, there are only a few articles in the literature demonstrating the benefit of HIFU with MR guidance for the palliative treatment of bone metastases [16, 22, 23].

Our study shows a significant decrease in patient pain after treatment by HIFU with MRI guidance (*p* < 0,05) at 1 week and at 1 month post procedure. Ten elderly patients were significantly relieved of their pain following the procedure. These patients had several co-morbidities and had exhausted maximum radiotherapeutic and analgesic treatment options for their painful bone metastases. All suffered intense inflammatory pain, often associated with mechanical pain severely decreasing their quality of life. The procedure indication was evaluated very carefully for each patient, as management of symptomatic bone metastases by HIFU depends largely on clinical symptoms and the degree of pain felt by patients. All the patients suffered from a symptomatic bone lesion. The association of bone marrow edema and enhancement of the lesion on the MRI performed before treatment indicated the concordance between clinical symptoms and imaging.

The aim of treatment was to obtain pain palliation and not total lesion destruction.

We believe that correct selection of patients is crucial. Indeed, we recorded one patient who experienced no relief of his pain and for whom the effectiveness of the denervation treatment was inconclusive, an observation which argues in favor of a multifaceted etiology of pain.

The feasibility of the HIFU procedure was 100% in our study. All procedures were performed under general anesthesia in order to ensure greater patient comfort since the interventions are lengthy and can be painful during sonications. This procedure can be performed under local anesthesia if necessary in case of contraindication to general anesthesia or, if the patient wishes, by applying good analgesic sedation before the procedure [22].

Hurwitz et al. recently reported the first completed phase III randomized trial investigating MRgFUS in patients with painful bone metastases. They showed a response rate of 64.3% in the MRgFUS arm compared with 20.0% in the placebo arm (*P* < .001) at 3 months [1]. Napoli et al. also showed a slightly higher response rate of 88.9% at 3 months post-treatment [24]. In the recent literature, an international consensus statement recognized MRgFUS as a safe and effective secondary treatment option in painful radiation-refractory bone metastases outside the spine [25]. MRgFUS may be considered in settings where primary therapeutic modalities—namely, radiotherapy—are contraindicated or are refused by the patient.

These therapeutic techniques appear to be complementary for oncology patients suffering from metastatic disease and can be used in combination for optimal antalgic and therapeutic effectiveness. Further evidence from large randomized control studies is needed to establish MRgFUS as a possible palliative treatment of bone metastases alongside other available therapeutic options [24].

HIFU seems to be a safe and effective treatment procedure as no immediate or delayed complications were observed in our study. To improve pain control during the intervention, patients were treated by direct approach with focused ablation of the periosteum as this is the most highly innervated component of mature bone tissue. In the literature, several possible ablation approaches have been described using HIFU: the “near-field approach” in patients with (partially) intact cortical bone at the targeted lesion, in which treatment cells are initially positioned behind the cortical bone; and the “direct approach” in which treatment cells are positioned on the bone/soft-tissue interface. Although both ablation approaches can induce thermal ablation of the bone/soft-tissue interface, the direct approach requires lower sonication energies compared to the near-field approach, thus minimizing the risk of thermal damage beyond the targeted volume [16]. HIFU treatment also seems to induce a decrease of serum immunosuppressive cytokines such as vascular endothelial growth factor (VEGF) in patients with solid tumors [26].

For optimal effectiveness, the benefit of the HIFU procedure should be estimated in terms of patient pain. However, this can be difficult as pain can be multifactorial in oncology patients. Bone metastases are painful current lesions in oncology patients. HIFU treatment enables us to relieve the pain felt by patients but does not promote bone consolidation, which is often needed in such fragile patients. In these circumstances, the benefits provided by a therapeutic association between different modalities such as cementoplasty, radiotherapy and immunotherapy becomes paramount [11, 27–29].

**Fig. 5:**
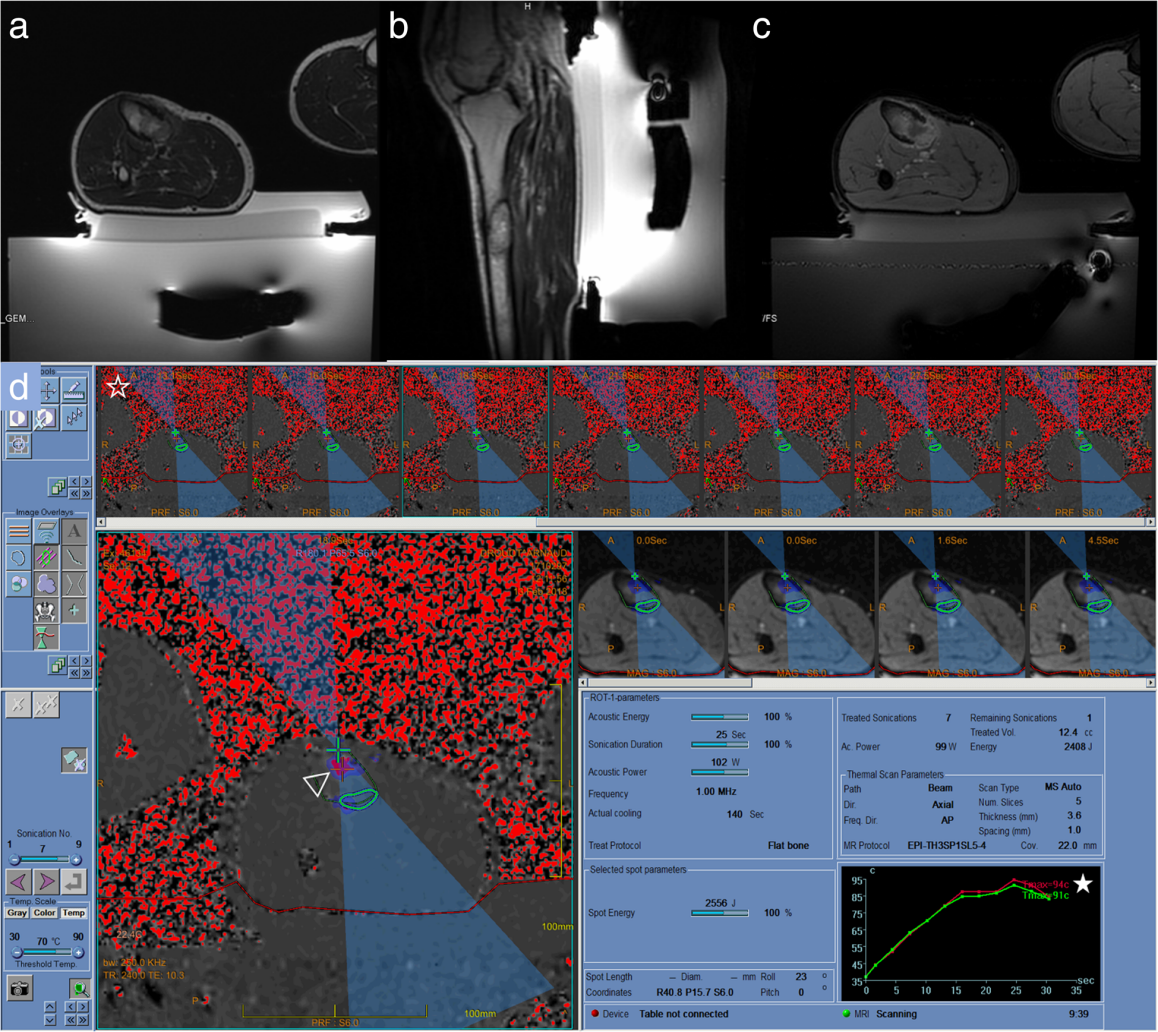
Patient 13. **a**, **b** Axial and Sagittal T2-weighted MR image showing a bone metastasis of the tibial diaphysis in front of the transducer. **c** Axial T1-weighted fat-suppressed with contrast MR image showing thermal destruction and necrosis inside the lesion. **d** Screen capture during sonication showing the thermal dose deposit (hollow arrowhead) inside the diaphysis. Thermal monitoring is performed using a map (empty star) and a graph (star). Temperature monitoring (star) shows a mean temperature of 91 °C at the end of this sonication

In MR-HIFU, image guidance is crucial for treatment planning and real-time temperature monitoring as lethal cell damage occurs when temperatures > 55 °C are maintained for longer than 1 s [30]. The measured temperatures represent an approximation of the true temperature of the bone/soft tissue interface, as only temperature differences occurring in aqueous soft-tissue adjacent to the cortical bone can be measured. The method is also sensitive to magnetic field disturbances and artifacts and partial volume may induce a degree of inaccuracy in temperature estimates [31–34] (Fig 5). We observed one patient who presented post-procedure superficial skin irritation, which we treated by anti-inflammatories for 5 days. During the long-term follow-up, four patients in our series died as a result of advanced metastatic disease.

Our study has several limitations. First, we did not compare our results with a control group treated conservatively or with radiation therapy alone. In fact, it was difficult not to treat demanding patients suffering intense pain and to whom we could propose a safe and efficient treatment option. Second, our patient sample was small and the follow-up period was relatively short; long-term clinical outcomes still need to be evaluated. MR-HIFU also has its limitations as the procedure is time-consuming and required general anesthesia in our study. The cost of the technique and its availability are also limiting factors. Indications and benefits of MR-guided HIFU in the treatment of bone metastases should be clearly defined for routine use by interventional radiologists, while new indications for the technique are currently under investigation [35, 36].

In conclusion, MR-guided HIFU seems to be a safe and efficient therapeutic option for patients suffering from bone metastases. The technique can be used alone or in combination with other treatments such as cementoplasty or radiotherapy for palliative treatment in metastatic disease. Patients should be carefully screened for optimal therapeutic effectiveness of the procedure. Future research should include a large well-designed cohort study with longer follow-up, in which patients with persistent metastatic bone pain are treated. The benefits versus risks ratio seems very positive, with a significant decrease in patient pain and the advantages of a non-invasive procedure. This non-invasive interventional radiology technique appears to be a promising additional tool for the management of patients in oncology.

## Abbreviations

FUs: Focused Ultrasound
HIFU: High-Intensity Focused Ultrasound
MR: Magnetic Resonance
MRI: Magnetic Resonance Imaging
OMEDD: Oral Morphine Equivalent Daily Dose
VAS: Visual Analog Scale
